# Milk fat globule epidermal growth factor-factor 8-derived peptide attenuates organ injury and improves survival in sepsis

**DOI:** 10.1186/s13054-015-1094-3

**Published:** 2015-10-28

**Authors:** Weng-Lang Yang, Archna Sharma, Fangming Zhang, Shingo Matsuo, Zhimin Wang, Haichao Wang, Ping Wang

**Affiliations:** Department of Surgery, Hofstra North Shore-LIJ School of Medicine, Manhasset, NY 11030 USA; Center for Translational Research, The Feinstein Institute for Medical Research, 350 Community Dr., Manhasset, NY 11030 USA; Laboratory for Emergency Medicine, The Feinstein Institute for Medical Research, Manhasset, NY 11030 USA; Present address: Department of Surgery II, Tokyo Women’s Medical University, Tokyo, Japan

## Abstract

**Introduction:**

Sepsis involves overwhelming inflammatory responses with subsequent immune-suppression that can lead to multiple organ dysfunction and ultimately death. Milk fat globule epidermal growth factor-factor 8 (MFG-E8) is a secretory protein found to have multiple biological activities against autoimmune and inflammatory diseases. MFG-E8 contains an Arg-Gly-Asp (RGD) motif involved in cell-cell and cell-matrix interactions. In sepsis, excessive neutrophils migration through endothelial cells and matrix to sites of inflammation results in organ damage. We hypothesized that MFG-E8-derived short peptides (MSP) flanking its RGD motif could provide protection against organ injury in sepsis.

**Methods:**

The differentiated human neutrophil-like HL-60 cells (dHL60) were incubated with a series of peptides flanking the RGD motif of human MFG-E8 for a cell adhesion assay to fibronectin or human pulmonary artery endothelial cells (PAECs). For the induction of sepsis, male C57BL/6 mice (20–25 g) were subjected to cecal ligation and puncture (CLP). Peptide MSP68 (1 mg/kg body weight) or normal saline (vehicle) was injected intravenously at 2 h after CLP. Blood and tissue samples were collected at 20 h after CLP for various measurements.

**Results:**

After screening, peptide MSP68 (VRGDV) had the highest inhibition of dHL-60 cell adhesion to fibronectin by 55.8 % and to PAEC by 67.7 %. MSP68 treatment significantly decreased plasma levels of organ injury marker AST by 37.1 % and the proinflammatory cytokines IL-6 and TNF-α by 61.9 % and 22.1 %, respectively after CLP. MSP68 improved the integrity of microscopic architectures, decreased IL-6 levels in the lungs by 85.1 %, and reduced apoptosis. MSP68 treatment also significantly reduced the total number of neutrophil infiltration by 61.9 % and 48.3 % as well as MPO activity by 40.8 % and 47.3 % in the lungs and liver, respectively, after CLP. Moreover, the number of bacteria translocated to mesenteric lymph nodes was decreased by 57 % with MSP68 treatment. Finally, the 10-day survival rate was increased from 26 % in the vehicle group to 58 % in the MSP68-treated group.

**Conclusions:**

MSP68 effectively inhibits excessive neutrophils infiltrating to organs, leading to moderate attenuation of organ injury and significantly improved survival in septic mice. Thus, MSP68 may be a potential therapeutic agent for treating sepsis.

## Introduction

Sepsis is defined as an infection-associated initial hyper-inflammatory and subsequent immune-suppressive response, which leads to multiple organ dysfunction, shock, secondary infections and lethality [1–3]. It is one of the most prevalent diseases and accounts for 20 % of all admission to intensive care units [4]. More than 800,000 people develop sepsis and septic shock annually, with an overall mortality of 30 % in the USA [5, 6]. Despite the tremendous efforts in advancing the understanding of sepsis progression and more than thirty failed clinical trials, there is still no effective drug available to treat this disease [7–10]. Undoubtedly, there is an urgent need to identify and develop a new class of therapeutic agents against sepsis and septic shock.

During the early stage of sepsis, neutrophils are recruited to the inflamed organs to contain and eradicate invading pathogens [11]. These activated neutrophils release proteolytic enzymes and reactive oxygen species. Although these released molecules help kill invading pathogens in sepsis, their excessive production disrupts the endothelial barrier and causes extravascular tissue damage, contributing significantly to multiple organ failure (MOF) and lethality [12–15]. Examination of autopsy specimens from patients with MOF reveals large-scale neutrophil infiltration of the lungs [16]. Thus, a therapeutic strategy designed to attenuate neutrophil infiltration has the potential to prevent organ injury and reduce mortality in sepsis [17, 18].

Human milk fat globule epidermal growth factor-factor 8 (MFG-E8) is a secretory 387-amino acid (aa) protein composed of a N-terminal cleavable signal peptide, one epidermal growth factor (EGF)-like domain, and two C-terminal discoidin domains which resemble the sequences of blood coagulation factors V and VIII [19]. The EGF-like domain contains an arginine-glycine-aspartate (RGD) motif which binds integrins of macrophages, while the discoidin domains bind phosphatidylserine, thus opsonizing apoptotic cells and promoting their engulfment by macrophages [19, 20]. Therefore, MFG-E8 facilitates the clearance of apoptotic cells by phagocytosis and reduces inflammatory responses, resulting in protection of animals from intestinal injury and sepsis [21, 22]. In addition, MFG-E8 directly inhibits proinflammatory cytokine release from immune cells via attenuation of the NF-κB pathway [23]. We have also identified that MFG-E8 is a novel regulator of neutrophil infiltration in acute lung injury [24, 25].

Neutrophil recruitment is a multiple-step process [26–28]. Under the normal condition, neutrophils roll along microvascular walls via low affinity interaction of selectins with endothelial cells. During inflammation, chemotactic factors induced by proinflammatory cytokines signal the recruitment of neutrophils into the sites of infection and/or injury. This leads to the activation of neutrophil integrins and the subsequent high-affinity binding of neutrophils to the activated endothelial cells in postcapillary venules. Under the influence of chemotactic gradients, neutrophils penetrate the endothelial layer and migrate through connective tissue to the sites of infection, where they finally congregate and adhere to extracellular matrix (ECM) components [29]. The binding of integrins on neutrophils to their ligands, especially containing the RGD sequence, is very crucial for this recruiting process [30]. Thus, we further examined whether the RGD motif in MFG-E8 was responsible for its activity in regulating neutrophil infiltration.

In the present study, we first screened a number of peptides derived from the sequence flanking RGD domain of human MFG-E8 by assessing their inhibition of neutrophil adhesion to fibronectin and endothelial cells. We then examined the effect of treatment with the identified peptide on the neutrophil trafficking and organ injury in septic mice induced by cecal ligation and puncture (CLP), a physiologically relevant model. This approach would not only dissect the specific domain of MFG-E8 in regulating neutrophil infiltration but also evaluate the potential of using MFG-E8-derived smaller peptides as a therapeutic strategy for treating sepsis.

## Methods

### Cell lines

Human promyelocytic leukemia cell line HL-60 and primary pulmonary artery endothelial cells (PAECs) were obtained from the American Type Culture Collection (ATCC, Manassas, VA, USA). HL-60 cells were cultured in RPMI medium (Invitrogen, Carlsbad, CA, USA) containing 10 % fetal bovine serum (FBS), 2 mM L-glutamine and 1 % penicillin and streptomycin. HL-60 cells were differentiated into neutrophil-like cells (dHL-60) by adding dimethyl sulfoxide at 12.7 μl/ml/million cells for 5 days. PAECs were cultured in vascular cell basal medium supplemented with endothelial cell growth kit-VEGF (ATCC).

### Cell adhesion assay

The dHL-60 cells were labeled with calcein AM (Life Technologies, Grand Island, NY, USA). 1.5 × 10^5^ labeled dHL-60 cells were added to 96-well plates coated with 10 μg/ml fibronectin (Life Technologies) or 10,000 PAECs/well in the presence of phosphate-buffered saline (PBS) or various concentrations (0.5 and 5 μg/ml) of synthesized small peptides derived from human MFG-E8 (GenScript, Piscataway, NJ, USA). The plates were incubated at 37 °C in 5 % CO_2_ for 2 h. Non-adherent cells were washed away with PBS and attached cells were detected using a fluorescence plate reader at 485 nm/530 nm.

### Mice

Male C57BL/6 mice (20 to 25 g) purchased from Taconic Biosciences (Albany, NY, USA) were used in all experiments at 8–12 weeks of age. These mice were housed in a temperature-controlled room on a 12-h light/dark cycle in the animal facility within the Feinstein Institute for Medical Research (Manhasset, NY, USA) and fed a standard laboratory diet. All experiments were performed in accordance with the recommendations in the Guide for the Care and Use of Laboratory Animals of the National Institutes of Health (Bethesda, MD, USA) and were approved by the Institutional Animal Care and Use Committee (IACUC) at the Feinstein Institute for Medical Research. All efforts were made to minimize suffering.

### Cecal ligation and puncture (CLP)

Sepsis was induced in mice using the CLP procedure. The mice were anesthetized by isoflurane inhalation, and the abdomen was shaved and cleaned with 10 % povidone iodine. A 1-cm to 2-cm midline incision was performed to expose the cecum, which was tightly ligated with a 4-0 silk suture at 1 cm from the tip. The ligated cecum was double punctured with a 22-gauge needle, gently squeezed to expel a small amount of feces from the perforation sites and returned to the peritoneal cavity. The laparotomy site was then closed with a 6-0 silk suture in two layers. The sham animals underwent the same procedure but the cecum was neither ligated nor punctured. The operated animals were resuscitated and 1 ml of normal saline was given by subcutaneous injection immediately after the surgery to improve dehydration. At 20 h after CLP or sham operation, mice were anesthetized and blood, liver, and lungs were collected. Blood samples were centrifuged at 3,000 *g* for 10 minutes to collect plasma. A section of lung tissue was preserved in formalin for histopathological analysis. The plasma and remainder of tissue samples were frozen immediately in liquid nitrogen, and stored at −80 °C until analysis. An additional set of experiments were performed for harvesting the livers, lungs and peritoneal fluids for leukocyte preparation 20 h post-CLP or sham. For the survival study, mice were subcutaneously administered 0.5 mg/kg of antibiotic PRIMAXIN (Merck, Whitehouse Station, NJ, USA) after CLP and were monitored for ten days to record survival.

### Administration of MSP68

Mice were allocated to three groups: sham, vehicle, or treatment. Two hours after CLP, a small incision on the neck was made and the internal jugular vein was exposed. Normal saline (vehicle) or MSP68 (GenScript) at a dose of 1 mg/kg body weight (BW) in 200 μl volume was delivered by injection using a 29G × 1/2″ U-100 insulin syringe (Terumo Medical Corporation, Elkton, MD, USA) through the jugular vein.

### Measurements of cytokine and organ injury marker

Interleukin (IL)-6 and tumor necrosis factor (TNF)-α levels in the plasma samples and lung tissues were quantified using mouse enzyme-linked immunosorbent assay (ELISA) kits (BD Biosciences, Franklin Lakes, NJ, USA). Plasma levels of aspartate aminotransferase (AST) were measured using a commercial assay kit (Pointe Scientific, Lincoln Park, MI, USA) according to the manufacturer’s instructions.

### Histologic examination

The lung tissues were fixed in 10 % formalin followed by paraffin embedding. The paraffin tissue blocks were cut into 5-μm sections, which were transferred to glass slides and stained with hematoxylin and eosin (H&E). Morphologic changes in the lung tissues were examined by light microscopy, documented by photographs and evaluated by two investigators in a blinded manner. Lung injury was assessed according to the following pathological features: (1) alveolar wall thickening, (2) vascular congestion, (3) intra-alveolar hemorrhage, (4) interstitial leukocyte infiltration, and (5) alveolar leukocyte infiltration. A semiquantitative scoring system based on the presence and severity of each of these features was used to designate scores from 0 to 3 for absent, mild, moderate, or severe injury and a cumulative total histology score was determined.

### TUNEL assay

A TUNEL (terminal deoxynucleotidyl transferase dUTP nick end-labeling) staining kit (Roche Diagnostics, Mannheim, Germany) was used to detect the presence of apoptotic cells in the lung sections according to the manufacturer’s instructions. The negative control was performed by incubating slides in the mixture containing only deoxynucleotidyl transferase. TUNEL-positive cells were counted in 10 microscopic fields per section under a fluorescence microscope (×200).

### Western blotting

Lung tissues were homogenized in lysis buffer (10 mM Tris-HCl, pH 7.5, 120 mM NaCl, 1 % NP-40, 1 % sodium deoxycholate, and 0.1 % SDS) containing a protease inhibitor cocktail (Roche Diagnostics) by sonication. Protein concentrations were determined by Bio-Rad Laboratories (Hercules, CA, USA) protein assay reagent. Lysates from lungs were fractionated on Bis-Tris gels (4–12 %) and transferred to nitrocellulose membrane. The membranes were then blocked with 5 % nonfat dry milk in Tris-buffered saline with Tween-20 and incubated with anti-cleaved caspase-3 (Cell Signaling Technology, Beverly, MA, USA) or anti-β-actin (Sigma-Aldrich, St Louis, MO, USA) antibodies. The bands were visualized using Pierce ECL 2 Western Blotting Substrate (Thermo Scientific, Southfield, MI, USA).

### Leukocyte suspension preparation from lungs and livers

Complete RPMI medium was prepared with 10 % FBS, 1 % Penn-Strep, 10 mM HEPES (4-(2-hydroxyethyl)-1-piperazineethanesulfonic acid), 2 mM L-Glutamine, and 5 × 10^−5^ M β-mercaptoethanol. Lungs were minced and digested in complete RPMI medium containing 100 U/ml collagenase type 1 (Worthington Biochemical, NJ, USA) and 20 U/ml DNase 1 (Roche Diagnostics) for 30 minutes at 37 °C in a shaker incubator. Lung leukocytes were isolated by density-gradient centrifugation (2,000 rpm, 20 minutes) using 44 % and 66 % Percoll (GE Healthcare Bio-Sciences AB, Uppsala, Sweden). Cells at the interface and below were collected, washed with PBS and resuspended in complete RPMI. Livers were minced and homogenized in PBS with 1 % FBS, followed by Percoll-gradient centrifugation similar to lungs for isolation of hepatic leukocytes. The total leukocytes numbers isolated from lungs and livers were determined by counting aliquots in a hemocytometer using the trypan blue exclusion method.

### Neutrophil staining and flow cytometry

Cells (1 × 10^6^) obtained from livers and lungs were pre-incubated with anti-mouse CD16/CD32 (93) to block FcγRII/III receptors. These cells were then stained for 30 minutes on ice with allophycocyanin (APC) conjugated anti-mouse Ly-6G (1A8) and peridinin chlorophyll protein-cyanine 5.5 (PerCP/Cy5.5) conjugated anti-mouse CD11b (M1/70) antibodies (Biolegend, San Diego, CA, USA) for staining neutrophils. The stained samples were acquired using the FACSVerse (BD Bioscience, San Jose, CA, USA). Forward light scatter or forward light scatter plus propidium iodide was used to exclude dead cells. The data were analyzed by FlowJo software (Tree Star, Ashland, OR, USA). The neutrophil numbers in livers and lungs were determined using the following formula:

$$ \mathrm{Neutrophil}\ \mathrm{numbers}=\mathrm{Total}\ \mathrm{leukocytes}\ \mathrm{recovered}\times \mathrm{Neutrophil}\ \mathrm{percentage}/100. $$

### Myeloperoxidase (MPO) activity assay

Lung tissues were homogenized in potassium phosphate buffer containing 0.5 % hexa-decyl-trimethyl-ammonium bromide by sonication. After centrifugation the supernatant was diluted in reaction solution containing o-dianisidine hydrochloride and hydrogen peroxide. The rate of change in optical density per minute was measured at 460 nm to calculate MPO activity.

### Oxidative burst measurement of peritoneal neutrophils

Spontaneous neutrophil oxidative burst activity was measured by flow cytometry by quantifying the conversion of dihydrorhodamine 123 (DHR) to rhodamine 123 as previously described [31]. Peritoneal cells were harvested from mice under isoflurane anesthesia. After aseptic preparation of the abdominal wall, 5 ml of sterile cold PBS with 1 % FBS was injected and aspirated into the peritoneal cavity twice. Cells in peritoneal washes were washed with PBS and counted. Peritoneal lavage cells were incubated with 50 μM of DHR (Life Technologies) at 37 °C for 30 minutes. DHR-stained cells were pre-incubated with anti-mouse CD16/CD32 (93) for 10 minutes followed by 15 minutes incubation with APC-anti-mouse Ly-6G (1A8) and PerCP/Cy5.5-anti-mouse CD11b (M1/70) antibodies (Biolegend) on ice. A minimum of 10,000 events were collected and analyzed using the FACSVerse.

### Bacterial counts

Bacterial counts were performed on mesenteric lymph nodes (MLN) which were aseptically harvested from mice under isoflurane anesthesia. Equal amounts of wet MLN were homogenized in sterile PBS at 4 °C and samples were serially diluted in sterile PBS. Log dilutions were plated on trypticase soy agar plates with 5 % sheep blood (BD Diagnostic Systems, Sparks, MD, USA), which were incubated at 37 °C for 24 h under aerobic conditions. The colony-forming units (CFU) were counted and the results were expressed as colony forming units (CFU) per mg of tissue (MLN).

### Statistical analysis

Data were analyzed using SigmaPlot11 graphing and statistical analysis software (Systat Software Inc., San Jose, CA, USA) and expressed as mean ± standard error of the mean (SEM). One-way analysis of variance (ANOVA) was used for comparing multiple groups with Student-Newman-Keuls’ (SNK) test. Student’s *t* test was used for two-group analysis. The Kaplan-Meier method was used for analyzing the survival data and comparisons between groups were done using the log-rank test. Differences in values were considered significant if *P* was <0.05.

## Results

### MSP68 inhibits neutrophil adhesion

**Fig. 1:**
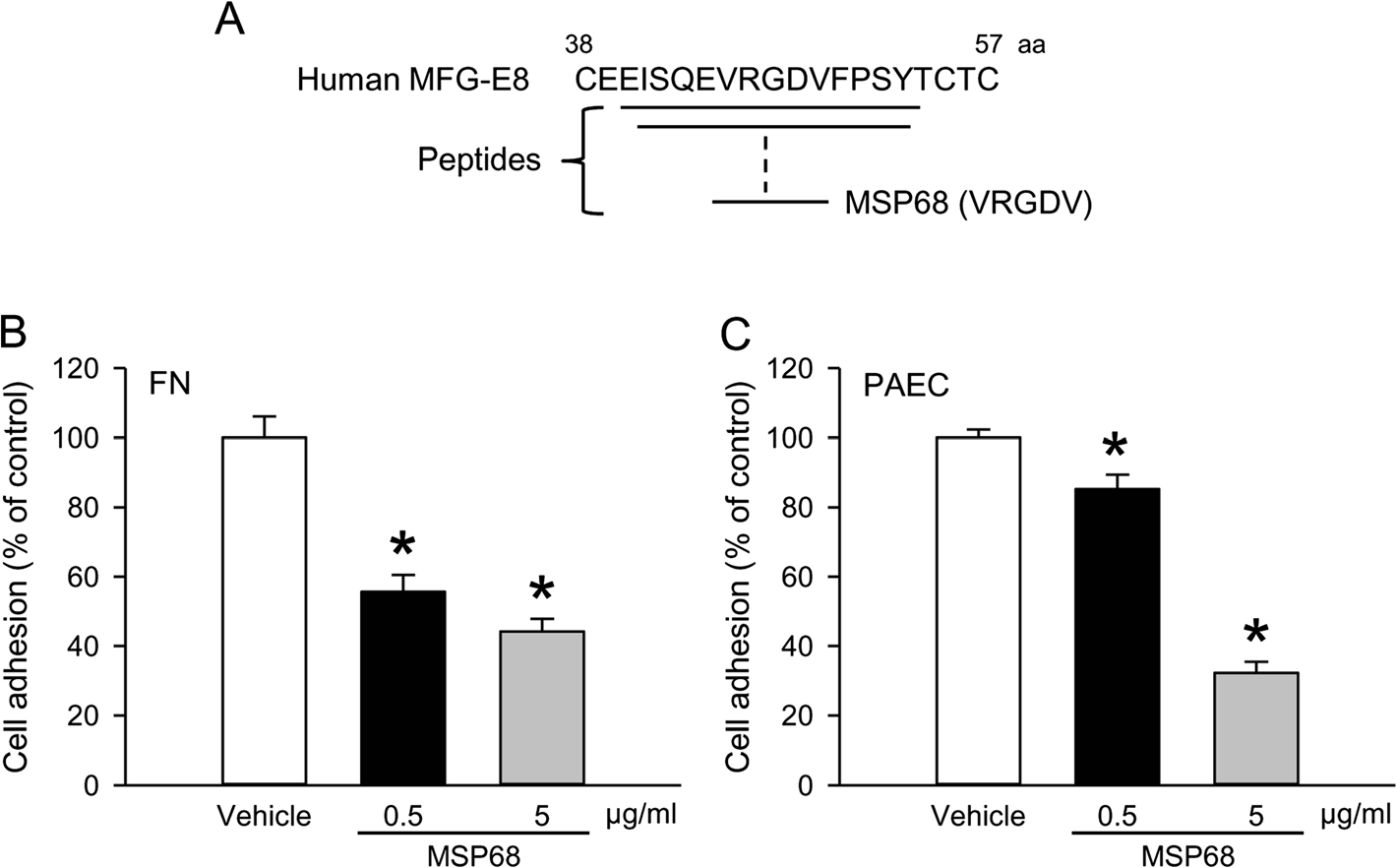
MFG-E8-derived short peptide 68 (MSP68) inhibits adhesion of neutrophils. **a** Schematic diagram showing a series of synthesized peptides flanking the arginine-glycine-aspartate (RGD) sequence of human MFG-E8 including the 5-aa peptide MSP68 with the valine-arginine-glycine-aspartate-valine (*VRGDV*) sequence. Calcein AM-labeled differentiated HL-60 cells were added to microtiter plates coated with fibronectin (*FN*) (10 μg/ml) (**b**) or 10,000 pulmonary artery endothelial cells (*PAECs*)/well (**c**) in the presence of vehicle (phosphate buffered saline (PBS)) or an indicated concentration of MSP68 and incubated at 37 °C for 2 h. Adhered dHL60 cells were detected using a fluorescence plate reader at 485 nm/530 nm. The fluorescence values for cells adhered to FN or PAEC in the presence of PBS are designated as 100 % adhesion. Data are expressed as mean ± standard error of the mean obtained from two independent experiments (n = 3/group) and compared by Student’s *t* test; *P <0.05 versus PBS

Cell adhesion plays an important role in the migration of neutrophils through the endothelium and extracellular matrix to sites of injury and inflammation and involves integrin binding. We synthesized a series of peptides (up to 15-mer) flanking the RGD sequence of human MFG-E8 (Fig. 1a) and extensively screened them by an in vitro cell adhesion assay to test their effect on neutrophil adhesion. Among them, a 5-aa peptide with the sequence valine-arginine-glycine-aspartate-valine (VRGDV), named MSP68, showed the most inhibition of the neutrophil adhesion to both fibronectin and endothelial cells. As seen in Fig. 1b, calcein AM-labeled dHL60 cells attached to fibronectin in the presence of PBS as 100 % adhesion, while MSP68 peptide significantly reduced the fibronectin adhesion of dHL60 cells by 44.3 % at 0.5 μg/ml and by 55.8 % at 5 μg/ml (Fig. 1b). Similarly, adhesion of dHL60 neutrophils to PAECs was reduced by 14.7 % and 67.7 % in the presence of 0.5 and 5 μg/ml of MSP68, respectively, compared to PBS (Fig. 1c).

### MSP68 treatment reduces organ injury and systemic inflammation in sepsis

**Fig. 2:**
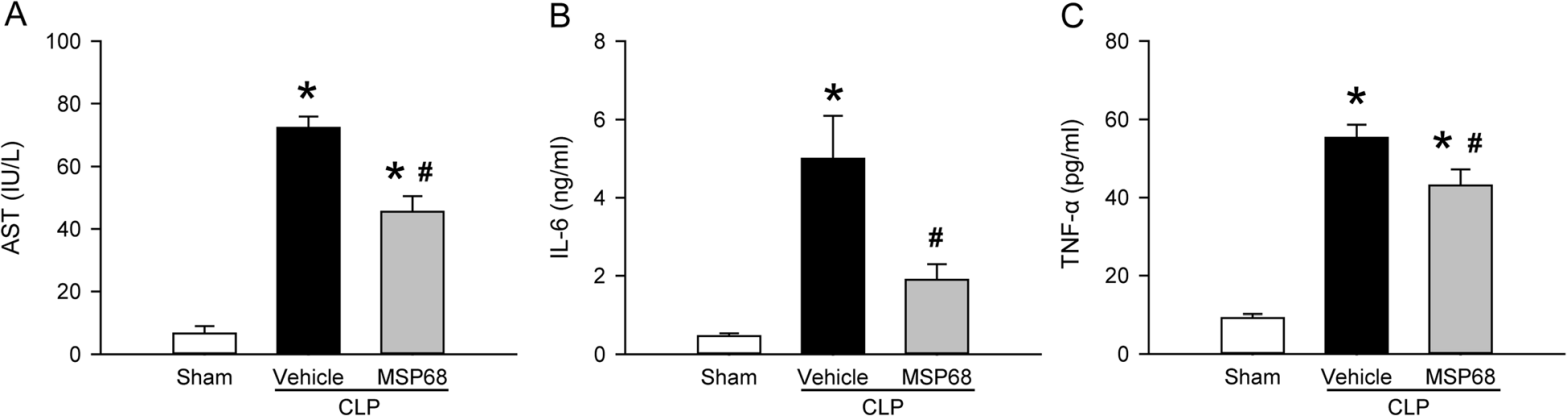
MFG-E8-derived short peptide 68 (*MSP68*) reduces organ injury after cecal ligation and puncture (*CLP*). Male C57BL/6 mice were sham-operated or subjected to CLP with injection of vehicle (normal saline) or MSP68 (1 mg/kg body weight) at 2 h after CLP. Blood samples were collected at 20 h after CLP to measure aspartate aminotransferase (*AST*) using the commercial assay kit (**a**), interleukin-6 (*IL-6*) (**b**) and tumor necrosis factor-α (*TNF-α*) (**c**) using enzyme-linked immunosorbent assay. Data are expressed as means ± standard error of the mean (n = 6 mice/group) and compared by one-way analysis of variance and Student–Newman–Keuls method; *P <0.05 versus sham and ^#^ *P* <0.05 versus vehicle

Sepsis is known to induce injury in multiple distant organs. Accordingly, the plasma levels of multiple organ injury marker, AST, were significantly increased by 10.8-fold in the vehicle group at 20 h after CLP, compared to the sham group (Fig. 2a). However, with MSP68 treatment, AST levels in these septic mice were 37.1 % lower than those in the vehicle-treated mice (Fig. 2a). The increase of plasma IL-6 levels has been correlated with the severity of sepsis [32, 33]. Indeed, IL-6 plasma levels were significantly increased by 10.7-fold in the vehicle group at 20 h after CLP, compared to the sham group (Fig. 2b). However, the IL-6 levels in the MSP68 group were 61.9 % lower than those in the vehicle group (Fig. 2b). Plasma levels of another proinflammatory cytokine, TNF-α, were also 5.9-fold higher in the vehicle group than the sham group and were decreased by 22.1 % on MSP68 treatment (Fig. 2c).

### MSP68 treatment attenuates sepsis-induced lung injury

**Fig. 3:**
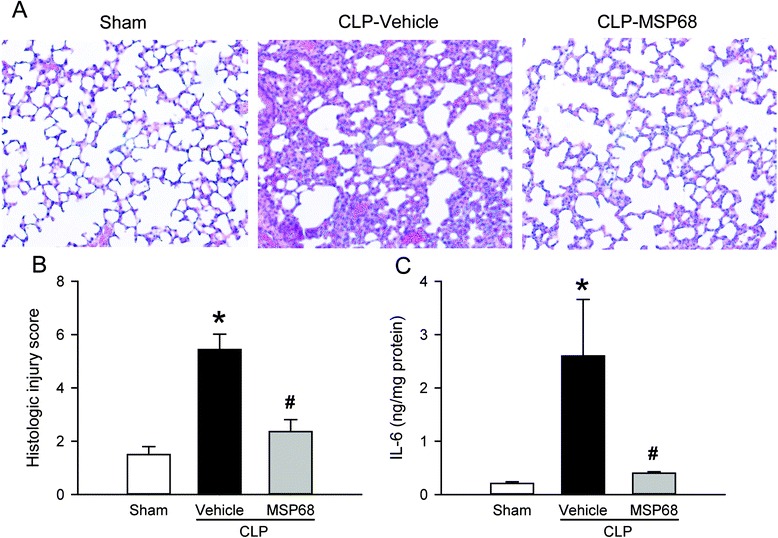
MFG-E8-derived short peptide 68 (*MSP68*) attenuates lung damage after cecal ligation and puncture (*CLP*). Male C57BL/6 mice were sham-operated or subjected to CLP with injection of vehicle (normal saline) or MSP68 (1 mg/kg body weight) at 2 h after CLP. The lung tissues were harvested at 20 h after CLP. **a** Sections of lung tissues were stained with hematoxylin and eosin, and examined under light microscopy. Representative images at original magnification × 200 are shown. **b** Histological lung injury scores from the above mentioned mice groups, determined as described in “Methods”, are shown. **c** Protein was extracted from the lung tissues to measure interleukin-6 (*IL-*6) levels by enzyme-linked immunosorbent assay. Data are expressed as means ± standard error of the mean (n = 4–6/group) and compared by one-way analysis of variance and Student–Newman–Keuls test; *P <0.05 versus sham and ^#^ *P* <0.05 versus vehicle

Acute lung injury is one of the most frequent complications of sepsis [34], so we examined the histological architecture of lungs at 20 h after CLP. The H&E staining revealed substantial morphological changes, including hemorrhage, edema, alveolar collapse, and infiltration of inflammatory leukocytes in the lung tissues of the vehicle-treated mice, compared to the sham mice (Fig. 3a). In contrast, the lung tissues from the MSP68-treated mice exhibited improved lung morphology with reduced microscopic deterioration, compared to the vehicle group (Fig. 3a). As quantified in Fig. 3b, the histological lung damage score in the vehicle group increased 3.6-fold in comparison with the sham group, while this score was significantly reduced by 56.5 % in the MSP68-treated mice. In addition, MSP68 treatment also significantly decreased the lung IL-6 protein level by 85.1 % in comparison with the vehicle group (Fig. 3c).

**Fig. 4:**
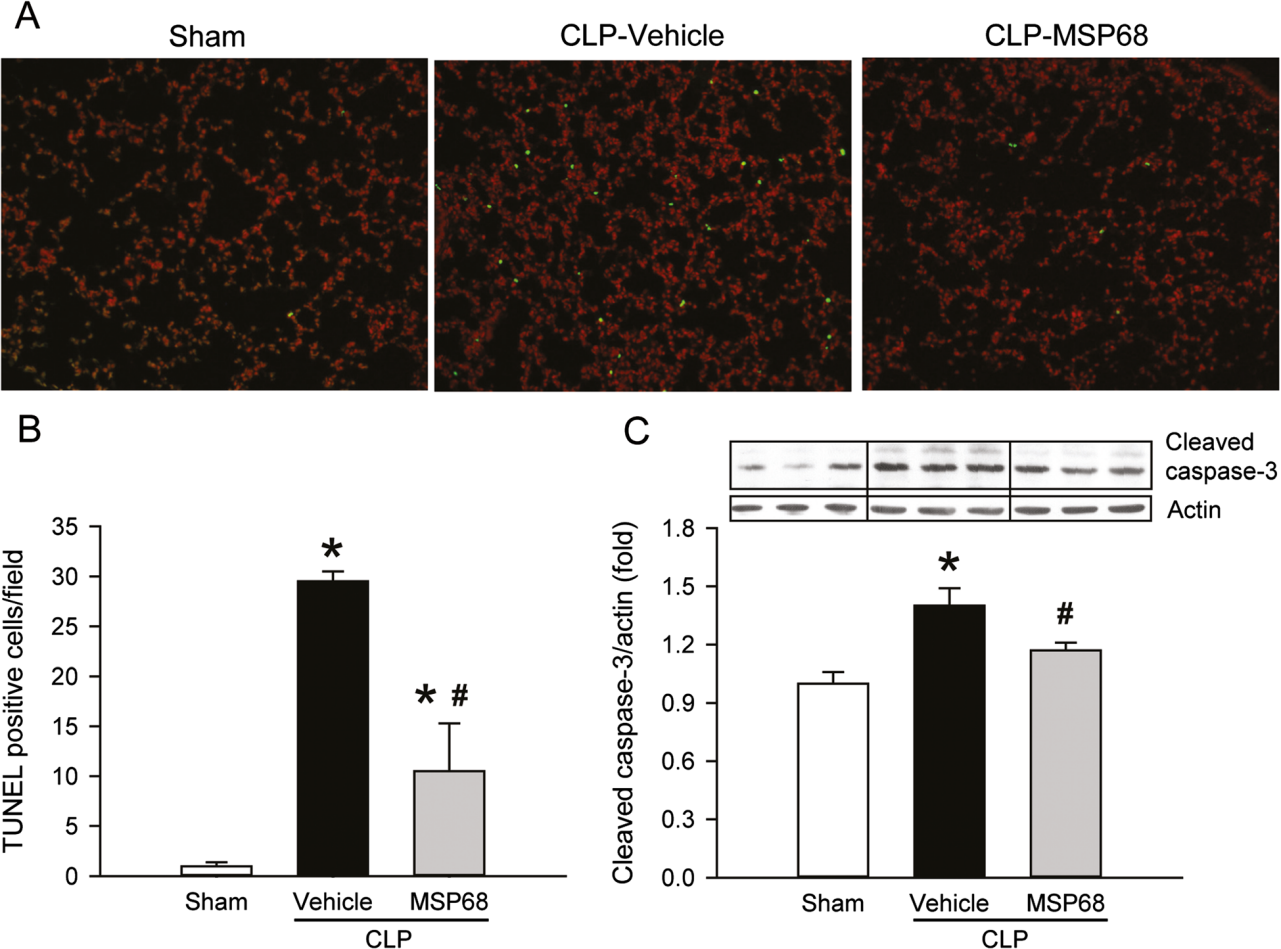
MFG-E8-derived short peptide 68 (*MSP68*) reduces apoptosis in the lungs after cecal ligation and puncture (*CLP*). Male C57BL/6 mice were sham-operated or subjected to CLP with injection of vehicle (normal saline) or MSP68 (1 mg/kg body weight) at 2 h after CLP. The lung tissues were harvested at 20 h after CLP. **a** Sections of lung tissues were stained with terminal deoxynucleotidyl transferase dUTP nick end-labeling (*TUNEL*) (*green fluorescent*), nuclear counterstained (*red fluorescent*), and examined under fluorescent microscopy. Representative images at original magnification × 200 are shown. **b** The numbers of apoptotic cells quantified from the TUNEL staining (averaged over 10 microscopic fields per mouse) are shown. **c** Protein was extracted from the lung tissues for western blotting. Representative blots for cleaved caspase-3 and loading β-actin control, and graph for their densitometric analysis are presented. Data are expressed as means ± standard error of the mean (n = 4–6/group) and compared by one-way analysis of variance and Student–Newman–Keuls test; *P <0.05 versus sham and ^#^ *P* <0.05 versus vehicle

Next, we performed a TUNEL assay on the lung tissues to investigate the effect of MSP68 treatment on lung apoptosis. The number of TUNEL-positive cells in the lung tissues of the vehicle group was markedly increased after CLP in comparison with the sham groups in which they were barely detectable (Fig. 4a). However, the number of apoptotic cells in the lung tissues of the MSP68-treated mice was significantly reduced by 64.4 % in comparison with the vehicle group (Fig. 4b). Similarly, the expression of cleaved caspase-3 in the lung tissues of the MSP68-treated mice was reduced by 17.7 % in comparison with that in the vehicle group (Fig. 4c). Taken together, our data suggest that MSP68 treatment improved CLP-induced lung damage and attenuated apoptotic cell death in the lungs.

### MSP68 treatment reduces neutrophil infiltration in the lungs and liver in sepsis

**Fig. 5:**
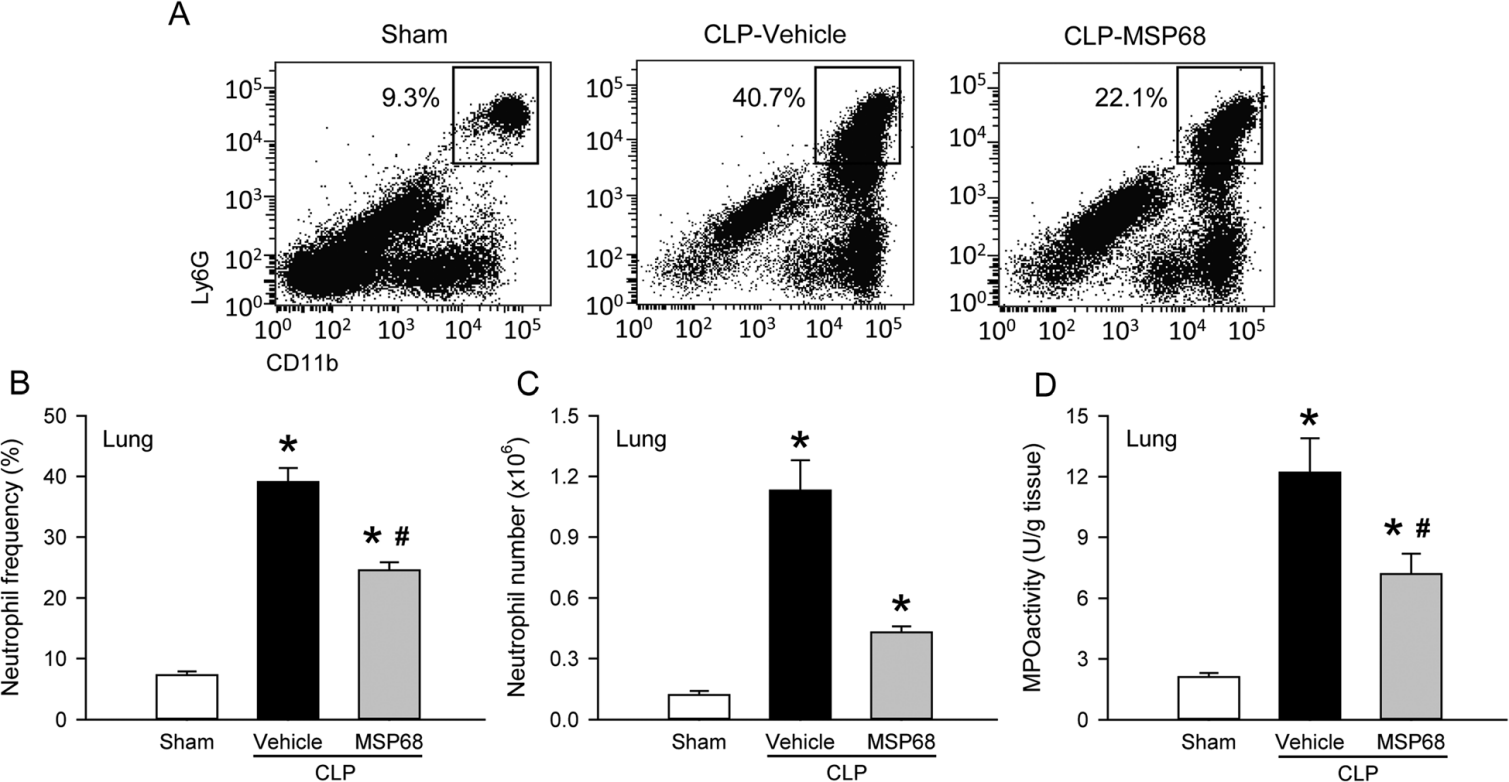
MFG-E8-derived short peptide 68 (*MSP68*) inhibits neutrophil infiltration in the lungs after cecal ligation and puncture (*CLP*). The lung tissues from sham, vehicle and MSP68-treated mice were harvested at 20 h after CLP. **a** Single cell suspensions of leukocytes were stained with allophycocyanin (APC)-anti-mouse Ly-6G and peridinin chlorophyll protein-cyanine (PerCP/Cy5.5)-anti-mouse CD11b, followed by flow cytometric analysis. Representative dot plots of surface Ly6G/CD11b expression on gated live lung leukocytes. Numbers adjacent to outlined areas show the percentage of Ly6G^+^CD11b^+^ neutrophils as indicated. The graphs show percentage (**b**) and total numbers of neutrophils in the lungs (**c**). **d** Lung tissues were homogenized and myeloperoxidase (*MPO*) activity was determined spectrophotometrically. Data are expressed as mean ± standard error of the mean (n = 4–6/group) and compared by one-way analysis of variance and Student–Newman–Keuls test; *P <0.05 versus sham and ^#^ *P* <0.05 versus vehicle

Neutrophil sequestration and activation in the lungs and liver are critical for causing acute injury in sepsis [14, 35]. To evaluate the neutrophil infiltration in the lungs and liver, we isolated leukocytes from these tissues and stained with Ly6G and CD11b surface markers to identify neutrophils. In the lungs, frequency of Ly6G^+^CD11b^+^ neutrophils in the vehicle group was increased by 5.3-fold in comparison with the sham group, while it was reduced by 37.0 % in the MSP68 group (Fig. 5a, b). Accordingly, the total number of lung neutrophils increased by 9.5-fold in the vehicle group, but reduced by 61.9 % with MSP68 treatment (Fig. 5c). Myleoperoxidase (MPO) activity correlates well with tissue neutrophil content and is used as a marker for neutrophil infiltration in the tissues. In consistence, there was a 5.9-fold increase of the lung MPO activity in the vehicle group in comparison with the sham group (Fig. 5d). With MSP68 treatment, the lung MPO activity was reduced by 40.8 % (Fig. 5d).

**Fig. 6:**
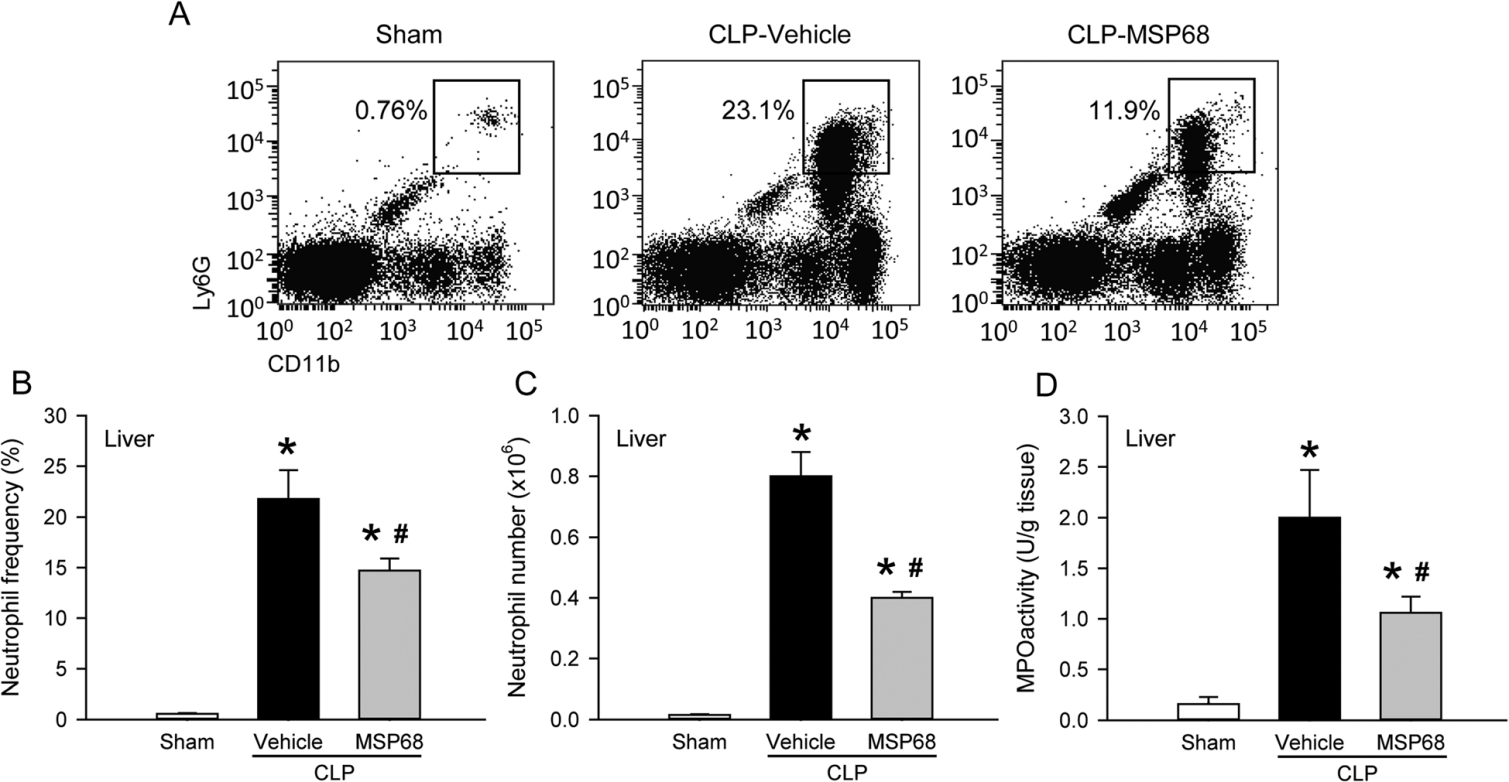
MFG-E8-derived short peptide 68 (*MSP68*) inhibits neutrophil infiltration in the liver after cecal ligation and puncture (*CLP*). The liver tissues from sham, vehicle and MSP68-treated mice were harvested at 20 h after CLP. **a** Single cell suspensions of leukocytes were stained with allophycocyanin (APC)-anti-mouse Ly-6G and peridinin chlorophyll protein-cyanine 5.5 (PerCP/Cy5.5)-anti-mouse CD11b, followed by flow cytometric analysis. Representative dot plots of surface Ly6G/CD11b expression on gated live liver leukocytes. Numbers adjacent to outlined areas show the percentage of Ly6G^+^CD11b^+^ neutrophils as indicated. The graphs show percentage (**b**) and total numbers (**c**) of neutrophils in the liver. **d** Liver tissues were homogenized and myeloperoxidase (*MPO*) activity was determined spectrophotometrically. Data are expressed as mean ± standard error of the mean (n = 4–6/group) and compared by one-way analysis of variance and Student–Newman–Keuls test; *P <0.05 versus sham and ^#^ *P* <0.05 versus vehicle

In the liver, the frequency of Ly6G^+^CD11b^+^ neutrophils was also significantly increased after CLP; however, it was reduced by 32.5 % in the MSP68 group in comparison with the vehicle group (Fig. 6a, b). Similar to the lungs, the total number of liver neutrophils increased 54.4-fold in the vehicle group, but reduced by 48.3 % with MSP68 treatment (Fig. 6c). In addition, the liver MPO activity in the vehicle group was increased 12.8-fold in comparison with the sham group, while it was reduced by 47.3 % in the MSP68 group (Fig. 6d). These results collectively show that MSP68 treatment attenuates CLP-induced lung and liver injury by reducing the associated neutrophil infiltration.

### MSP68 treatment does not hinder oxidative function of peritoneal neutrophils in sepsis

**Fig. 7:**
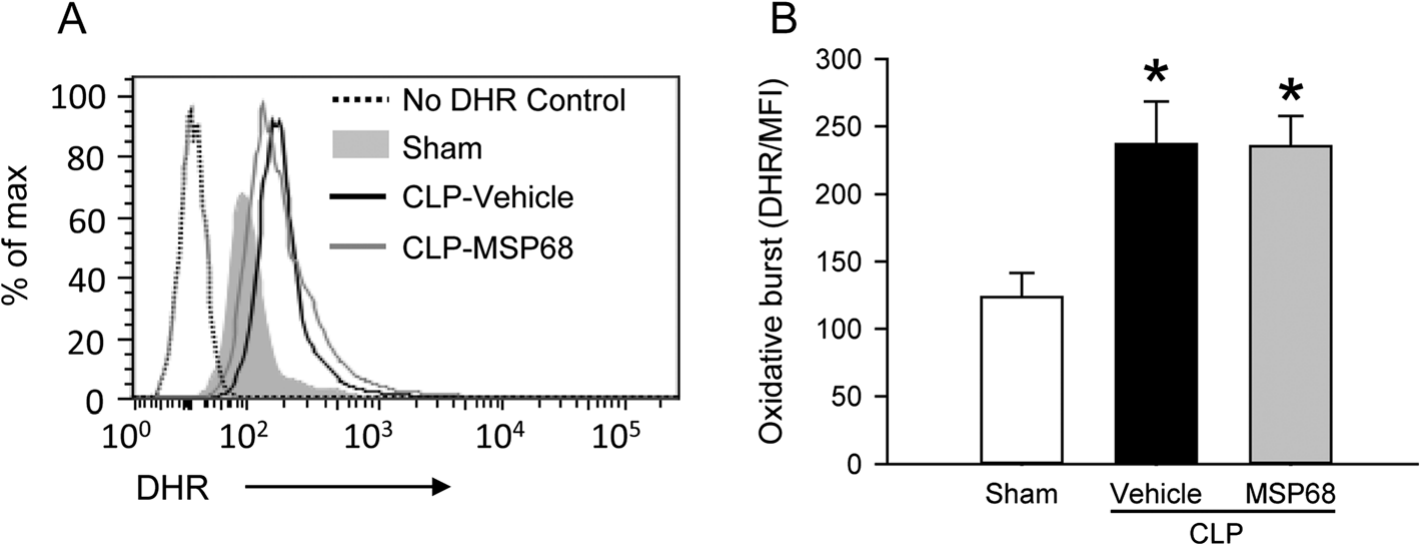
MFG-E8-derived short peptide 68 (*MSP68*) does not affect oxidative burst activity of neutrophils after cecal ligation and puncture (*CLP*). Peritoneal fluids from sham, vehicle and MSP68-treated mice were harvested at 20 h after CLP and processed to obtain single cell suspensions. Isolated peritoneal cells were incubated with 50 μM of *dihydrorhodamine 123* (DHR) at 37 °C for 30 minutes, followed by staining with allophycocyanin (APC)-anti-mouse Ly-6G and peridinin chlorophyll protein-cyanine 5.5 (PerCP/Cy5.5)-anti-mouse CD11b. Flow cytometry was done to quantify the fluorescence from DHR. **a** Representative histogram overlays show the flow cytometric analysis of DHR on the gated peritoneal neutrophils. **b** The graphs show the spontaneous neutrophil oxidative burst activity as the mean fluorescence intensity (*MFI*) of DHR on gated peritoneal neutrophils. Data are expressed as mean ± standard error of the mean (n = 3/group) and compared by one-way analysis of variance and Student–Newman–Keuls test; *P <0.05 versus sham

Neutrophils generate reactive oxygen species (ROS) during phagocytosis, which can result in damage of host tissues. On the other hand, this functional response, termed oxidative burst, is also part of a powerful germ-killing system of neutrophils [36]. As we noticed decreased neutrophil infiltration in the lungs and liver after CLP, we examined whether MSP68 treatment affected the oxidative function of neutrophils needed for eliminating bacteria. Representative histogram overlays showed increased DHR fluorescence in peritoneal neutrophils from the vehicle and MSP68 groups, compared to the sham group (Fig. 7a). However, there was no difference in their spontaneous oxidative burst activity between the MSP68-treated and vehicle groups at 20 h after CLP (Fig. 7b). These data showed that oxidative function of peritoneal neutrophils remains intact after MSP68 treatment in septic mice.

### MSP68 treatment prevents bacterial translocation in sepsis

**Fig. 8:**
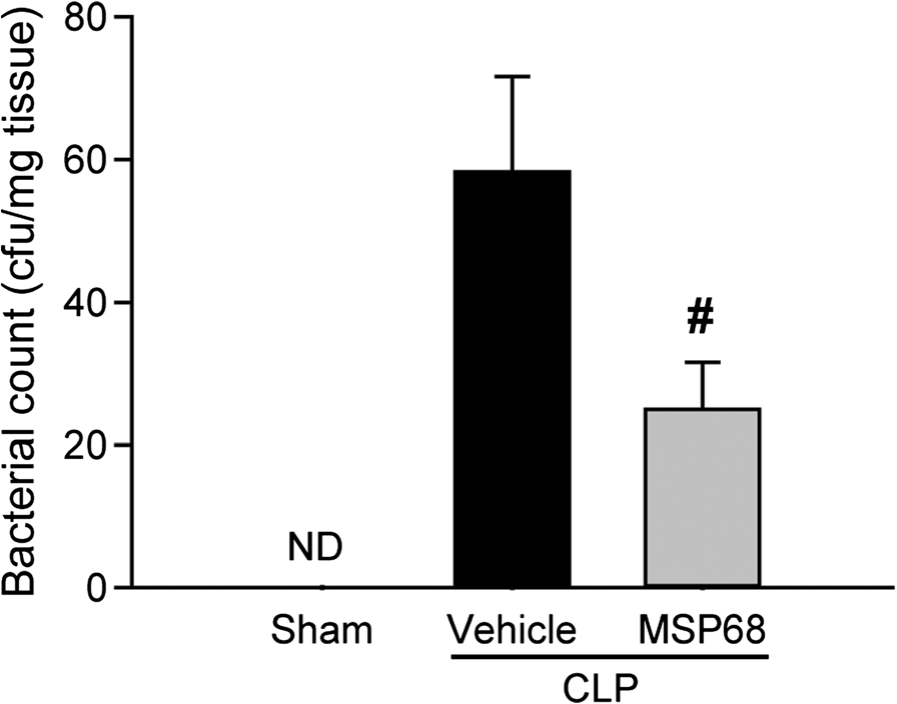
MFG-E8-derived short peptide 6 (*MSP68*) prevents bacterial translocation to mesenteric lymph nodes (MLN) after cecal ligation and puncture (*CLP*). MLN were aseptically collected at 20 h after CLP from sham, vehicle and MSP68-treated mice and homogenized in sterile PBS. The serially diluted MLN suspensions were plated, and bacterial colony forming units (*cfu*) were calculated. Data are expressed as mean ± standard error of the mean (n = 4–6/group) and compared by Student’s *t* test; ^#^ *P* <0.05 versus vehicle. *ND* not detectable

Bacterial translocation is the migration of viable bacteria from the gastrointestinal tract to normally sterile extra-intestinal sites, such as the mesenteric lymph nodes (MLN), which results in worsening of the outcome of sepsis [37]. To investigate the effect of MSP68 treatment on the extent of bacterial translocation in sepsis, we collected MLN from sham, vehicle and MSP68-treated mice at 20 h after CLP. Sterile suspensions from homogenized MLN were cultured for 24 h to assess the bacterial counts. No bacteria were detected in the MLN cultures from the sham group, whereas bacterial colonies were found in both vehicle and MSPP68 groups. Colony counts were significantly reduced by 57 % in the MLN cultures from MSP68-treated mice, compared to the vehicle group (Fig. 8).

### MSP68 treatment improves the survival of septic mice induced by CLP

**Fig. 9:**
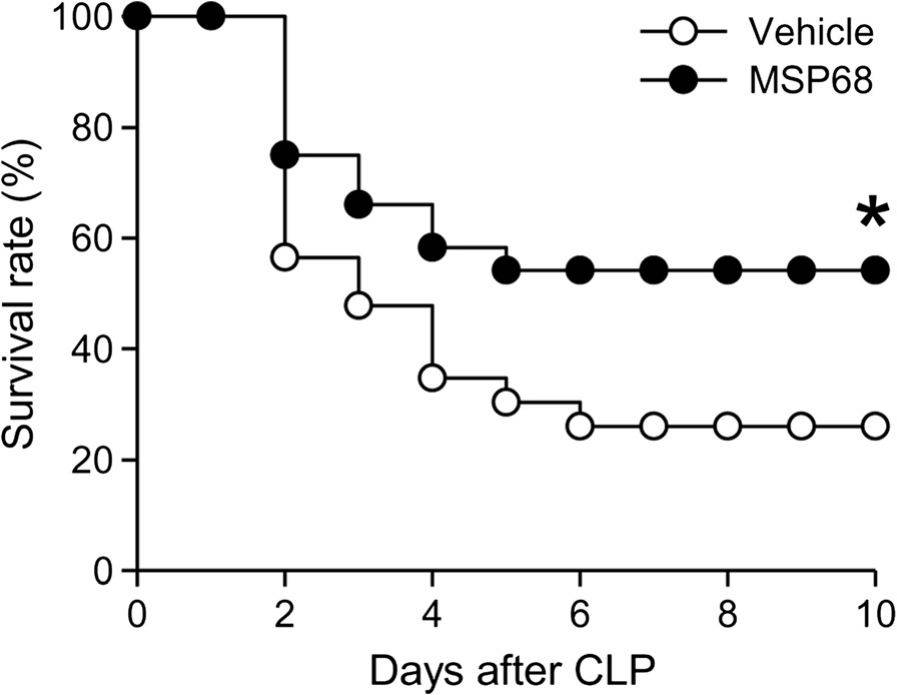
MFG-E8-derived short peptide 6 (*MSP68*) improves the survival in cecal ligation and puncture (*CLP*)-induced sepsis. Mice were subjected to CLP followed by a single subcutaneous injection of 0.5 mg/kg antibiotic. At 2 h after CLP, mice were injected with normal saline as vehicle (n = 23) or 1 mg/kg of MSP68 (n = 24) through the jugular vein and survival was recorded for 10 days. The survival rate was estimated by the Kaplan–Meier method and compared using the log-rank test; *P = 0.05 versus vehicle

To explore the long-term effect of MSP68 treatment on the septic mice, we performed a 10-day survival study on mice injected with vehicle or 1 mg/kg BW of MSP68 at 2 h after CLP. As shown in Fig. 9, the survival rate after CLP in vehicle-treated animals was 56.5 % on day 2, and gradually decreased to 26 % on days 6–10. Administration of MSP68 significantly improved the 10-day survival rate increasing it to 54 % with improvement in survival starting as early as day 2 (Fig. 9).

## Discussion

Despite advances in the management of sepsis patients, a large number of those patients die from the ensuing septic shock and MOF. Thus, there is an urgent unmet medical need for a novel and effective therapy for sepsis. Excessive recruitment of activated neutrophils into various tissues is a major contributing factor for causing organ injury in sepsis. Previous study from our lab has demonstrated the beneficial effect of MFG-E8 in reducing inflammatory responses and organ injury, and improving survival in a CLP-induced sepsis model [22, 38–41]. Earlier, we have also discovered a novel property of MFG-E8 in inhibiting the infiltration of activated neutrophils [24, 25], in addition to its activity on enhancing the clearance of apoptotic cells [42, 43]. However, the significance of the contribution of the MFG-E8 RGD motif in interfering with neutrophil infiltration has not been addressed.

In this study, we screened numbers of human MFG-E8-derived peptides flanking the RGD sequence, which could be involved in its binding to integrins. We have identified a short peptide, named MSP68, which strongly inhibits neutrophil adhesion to fibronectin and endothelial cells. We have demonstrated that post-treatment with MSP68 significantly reduces the inflammatory response by decreasing the IL-6 levels systemically and locally, and attenuates organ damage in septic mice. We have also shown that MSP68 treatment improves the integrity of the lung tissue and decreases lung apoptosis. Furthermore, MSP68 treatment effectively inhibits neutrophil infiltration to the organs, demonstrated by the reduction in the number of Ly6G^+^CD11b^+^ neutrophils and MPO activity in the lungs and liver of septic mice. However, MSP68 treatment does not hinder the oxidative function of peritoneal neutrophils in killing bacteria in septic mice. In fact, it decreases the bacterial translocation in septic mice. Finally, MSP68 treatment markedly increased 10-day survival of mice with CLP-induced sepsis.

Integrins are heterodimeric proteins composed of non-covalently associated α and β chains and are located on the cell surface to mediate cell-cell and cell-matrix interactions [30]. Twenty-four different integrins have been identified in humans [30]. Integrins α_L_β_2_ and α_M_β_2_ are the most important for neutrophils to bind to intercellular adhesion molecule 1 on endothelial cells. Integrin α_4_β_1_ is also used by neutrophils for binding to endothelial vascular cell adhesion molecule. Other integrins α_5_β_1_, α_IIb_β_1_, and α_V_β_3_, interact with several extracellular matrix components, such as fibronectin and collagen, by recognizing the tripeptide sequence RGD [30]. Neutrophils also bind to fibronectin and endothelial cells via integrins β_2_ which can also recognize RGD [30]. The relative affinity and specificity of the RGD peptides to different adhesion receptors have been indicated to be influenced by their flanking sequences and conformation [44]. The MSP68 (VRGDV) peptide demonstrated here shows interference with a broad spectrum of integrins to interact with various adhesion receptors, resulting in inhibition of neutrophil adhesion to fibronectin and endothelial cells. The specificity of physical interaction between MSP68 and different adhesion receptors is under investigation in our laboratory.

Effectiveness of synthetic RGD peptides in attenuating the lipopolysaccharide (LPS)-, mechanical ventilation- and intestinal ischemia-reperfusion (I/R)- induced lung injury has been demonstrated previously [45–47]. Their protective effects are attributed to inhibition of the infiltration of neutrophils and macrophages in the lung tissue and to the levels of TNF-α, IL-6, monocyte chemoattractant protein-2 and matrix metalloproteinase-9 [45–47]. Specifically, the synthetic RGD peptides inhibit integrin α_v_β_3_-mediated signaling in LPS- and mechanical ventilation-induced lung injury models [45, 46]. RGD-containing peptides have also been shown to protect the liver from cold I/R injury in steatotic liver transplants by inhibiting the recruitment of macrophages and neutrophils via blockade of integrin α_5_β_1_-fibronectin interactions and by reducing the expression of pro-inflammatory mediators, such as inducible nitric oxide synthase and interferon-gamma and matrix metalloproteinase-9 [48, 49].

Similar to our findings, a recent study has also reported a protective effect of synthetic RGD peptides in sepsis-induced ALI [50]. That study shows that administration of RGD peptides reduces systemic as well as lung levels of TNF-α and IL-6, and improves histopathology of lung tissue; however, the involvement of neutrophils has not been investigated [50]. By applying MSP68, we have further demonstrated the RGD peptide effectively decreases the total number of neutrophils infiltrating the lungs and liver, which is associated with reducing the severity of organ injury in septic mice. However, we have only examined architectural changes in the lungs with regard to organ injury in this study. Whether MSP68 treatment can also alleviate the sepsis-induced impairment of pulmonary and liver function needs further investigation. Also, sepsis commonly causes acute kidney injury which has not been the focus of this study. Further studies are needed to determine the effect of MSP68 treatment on the sepsis-induced acute kidney injury. Correspondingly, we have previously shown that administration of recombinant murine MFG-E8 can decrease neutrophil migration in LPS-induced acute lung injury by disrupting the interaction with integrin α_v_β_3_ [24]. These studies further support the approach of targeting integrin-mediated signaling using RGD-based peptides to control neutrophil infiltration into the organs, which subsequently induce organ injury in sepsis.

In addition to our data showing that MSP68 reduces excessive organ infiltration of neutrophils by inhibiting their adhesion, there are other mechanisms by which the decreased neutrophil content in the studied organs could be explained. Interestingly, integrin-mediated adhesion of neutrophils with endothelial cells/matrix has been reported to interfere with neutrophil life span, delaying their spontaneous apoptosis [51]. As prolonged neutrophil survival is part of the pathogenesis of sepsis, there is a possibility that MSP68 could be reducing the life span of neutrophils resulting into less accumulation in the organs. Whether or not MSP68 alters the phagocytic function of neutrophils under normal as well as septic conditions also remains to be investigated. A future study is granted to delineate the different possible mechanisms by which MSP68 can exert its positive effect on improving neutrophil-related organ injury in sepsis.

Maintenance of normal intestinal epithelial structure and function is important in preventing movement of bacteria and increased intestinal permeability has been reported in sepsis [52]. Bacterial translocation is associated with an increased risk of complications, MOF, or even mortality in critically ill sepsis patients [37]. We have previously shown that recombinant murine MFGE-8 treatment reduces the bacterial translocation to the MLN after gut I/R injury [53]. The current study shows that a 5-aa short peptide derived from human MFGE-8 sequence flanked RGD motif is also capable of lowering the bacterial translocation to MLN in a mouse model of CLP-induced sepsis. However, we have only counted viable bacteria in the MLN and not measured products from dead bacteria. Sepsis is known to impair the immune system, which is unable to effectively clear bacteria and bacterial products. Therefore, increase in levels of translocated viable bacteria and bacterial products could result from combined dysfunction of the intestinal barrier and the immune system, and not necessarily from increased intestinal permeability alone. Nevertheless, increased viable bacteria in MLN indicate increased risk of progression of infection in sepsis. As MSP68 does not affect the oxidative burst activity of peritoneal neutrophils, its reduction of bacterial translocation is most likely an indirect effect. In agreement with our observation, another study showed that treatment with synthetic RGD peptides modulated inflammation in sepsis but had no effect on peritoneal fluid bacterial load [50].

The CLP mouse model used in this study showed 74 % mortality in the vehicle group by day 10 which went down to 46 % after MSP68 treatment. However, we do recognize the limitations associated with the use of CLP models. CLP tends to have variable mortality rates based on differences between the strains and age of the animals, length of the ligated cecum, needle size and number of punctures, fluid resuscitation, and antibiotic treatment [8]. Also in this study, we administered MSP68 at 1 mg/kg BW and showed its beneficial effects in sepsis. Recent reports have used synthetic RGD peptides at 5 mg/kg BW to show significant inhibition of neutrophils infiltrating the lungs in the LPS-, intestinal I/R- and CLP-induced ALI model [45, 47, 50]. We therefore speculate that administration of MSP68 at the 5 mg/kg dose may increase its protective effect in CLP-induced sepsis. On the other hand, much higher doses may lessen its protective effect because neutrophils are necessary for the killing of invading pathogens. In the current study, we have administered normal saline as vehicle to be used as a baseline for comparing the effect of MSP68 administration on the outcome of sepsis. Scrambled peptide sequence used as control would have further assured that the effects are MSP68-peptide-specific and not potential unidentified off-target effects.

## Conclusions

In conclusion, data provided in this study identifies an MFG-E8 derived short-peptide, MSP68, which inhibits neutrophil adhesion. Treatment with MSP68 moderately attenuates sepsis-induced organ injury and systemic inflammation. In particular, MSP68 improves the histopathology of the lung tissue. MSP68 does so by effectively inhibiting the sepsis-induced excessive neutrophil infiltration into the lungs and liver. MSP68 treatment also reduces bacterial translocation and prolongs the survival of mice after sepsis. Thus, MSP68 may be a potential therapeutic agent for treating sepsis.

## Key messages

- A short peptide, named MSP68, derived from milk fat globule epidermal growth factor-factor 8 (MFG-E8) is identified as a novel inhibitor of neutrophil adhesion
- Post-treatment with MSP68 in CLP-induced sepsis there are reduced systemic levels of organ injury marker and pro-inflammatory cytokines IL-6 and TNF-α
- MSP68 treatment improves CLP-induced histopathology along with attenuating inflammation and apoptotic cell death in the lungs, which is associated with inhibition of the excessive neutrophil infiltration in the organs of septic animals
- MSP68 treatment does not hinder with the oxidative function of peritoneal neutrophils but reduces CLP-induced bacterial translocation
- MSP68 treatment improves the survival of mice with CLP-induced sepsis

## Abbreviations

aa: Amino acid
ALI: Acute lung injury
ANOVA: One-way analysis of variance
AST: Aspartate aminotransferase
BW: Body weight
CFU: Colony-forming units
CLP: Cecal ligation and puncture
dHL-60: Differentiated HL-60 cells
DHR: Dihydrorhodamine 123
ELISA: Enzyme-linked immunosorbent assay
FBS: Fetal bovine serum
H&E: Hematoxylin and eosin
HEPES: 4-(2-hydroxyethyl)-1-piperazineethanesulfonic acid
HL-60: Human promyelocytic leukemia cell line
IL: Interleukin
I/R: Ischemia-reperfusion
LPS: Lipopolysaccharide
MFG-E8: Milk fat globule epidermal growth factor-factor VIII
MFI: Mean fluorescence intensity
MLN: Mesenteric lymph nodes
MPO: Myleoperoxidase
MSP68: MFG-E8-derived short peptide 68
PAEC: Pulmonary artery endothelial cells
PBS: Phosphate-buffered saline
PI: Propidium iodide
RGD: Arginine-glycine-aspartate
SEM: Standard error of the mean
SNK: Student-Newman-Keuls’ test
TNF: Tumor necrosis factor
TUNEL: Terminal deoxynucleotidyl transferase dUTP nick end-labeling
VRGDV: Valine-arginine-glycine-aspartate-valine

## References

[1] Xiao W, Mindrinos MN, Seok J, Cuschieri J, Cuenca AG, Gao H (2011). A genomic storm in critically injured humans. J Exp Med.

[2] Hotchkiss RS, Monneret G, Payen D (2013). Sepsis-induced immunosuppression: from cellular dysfunctions to immunotherapy. Nat Rev Immunol.

[3] Shankar-Hari M, Deutschman CS, Singer M (2015). Do we need a new definition of sepsis?. Intensive Care Med.

[4] Levy MM, Dellinger RP, Townsend SR, Linde-Zwirble WT, Marshall JC, Bion J (2010). The Surviving Sepsis Campaign: results of an international guideline-based performance improvement program targeting severe sepsis. Crit Care Med.

[5] Gaieski DF, Edwards JM, Kallan MJ, Carr BG (2013). Benchmarking the incidence and mortality of severe sepsis in the United States. Crit Care Med.

[6] Angus DC, van der Poll T (2013). Severe sepsis and septic shock. N Engl J Med.

[7] Marshall JC (2008). Sepsis: rethinking the approach to clinical research. J Leukoc Biol.

[8] Ulloa L, Brunner M, Ramos L, Deitch EA (2009). Scientific and clinical challenges in sepsis. Curr Pharm Des.

[9] Opal SM, Dellinger RP, Vincent JL, Masur H, Angus DC (2014). The next generation of sepsis clinical trial designs: what is next after the demise of recombinant human activated protein C?*. Crit Care Med.

[10] Seam N, Suffredini AF (2007). Mechanisms of sepsis and insights from clinical trials. Drug Discov Today Dis Mech.

[11] Haziot A, Hijiya N, Gangloff SC, Silver J, Goyert SM (2001). Induction of a novel mechanism of accelerated bacterial clearance by lipopolysaccharide in CD14-deficient and Toll-like receptor 4-deficient mice. J Immunol.

[12] Lee WL, Downey GP (2001). Neutrophil activation and acute lung injury. Curr Opin Crit Care.

[13] Abraham E (2003). Neutrophils and acute lung injury. Crit Care Med.

[14] Ware LB (2006). Pathophysiology of acute lung injury and the acute respiratory distress syndrome. Semin Respir Crit Care Med.

[15] Herzig DS, Driver BR, Fang G, Toliver-Kinsky TE, Shute EN, Sherwood ER (2012). Regulation of lymphocyte trafficking by CXC chemokine receptor 3 during septic shock. Am J Respir Crit Care Med.

[16] Brealey D, Singer M (2000). Multi-organ dysfunction in the critically ill: epidemiology, pathophysiology and management. J R Coll Physicians Lond.

[17] Lewis SM, Khan N, Beale R, Treacher DF, Brown KA (2013). Depletion of blood neutrophils from patients with sepsis: treatment for the future?. Int Immunopharmacol.

[18] Mondrinos MJ, Kennedy PA, Lyons M, Deutschman CS, Kilpatrick LE (2013). Protein kinase C and acute respiratory distress syndrome. Shock.

[19] Hanayama R, Tanaka M, Miwa K, Shinohara A, Iwamatsu A, Nagata S (2002). Identification of a factor that links apoptotic cells to phagocytes. Nature.

[20] Andersen MH, Graversen H, Fedosov SN, Petersen TE, Rasmussen JT (2000). Functional analyses of two cellular binding domains of bovine lactadherin. Biochemistry.

[21] Bu HF, Zuo XL, Wang X, Ensslin MA, Koti V, Hsueh W (2007). Milk fat globule-EGF factor 8/lactadherin plays a crucial role in maintenance and repair of murine intestinal epithelium. J Clin Invest.

[22] Matsuda A, Jacob A, Wu R, Zhou M, Nicastro JM, Coppa GF (2011). Milk fat globule-EGF factor VIII in sepsis and ischemia-reperfusion injury. Mol Med.

[23] Aziz M, Jacob A, Matsuda A, Wu R, Zhou M, Dong W (2011). Pre-treatment of recombinant mouse MFG-E8 downregulates LPS-induced TNF-alpha production in macrophages via STAT3-mediated SOCS3 activation. PLoS One.

[24] Aziz M, Matsuda A, Yang WL, Jacob A, Wang P (2012). Milk fat globule-epidermal growth factor-factor 8 attenuates neutrophil infiltration in acute lung injury via modulation of CXCR2. J Immunol.

[25] Aziz M, Yang WL, Corbo LM, Chaung WW, Matsuo S, Wang P (2015). MFG-E8 inhibits neutrophil migration through alphavbeta3-integrin-dependent MAP kinase activation. Int J Mol Med.

[26] Ley K, Laudanna C, Cybulsky MI, Nourshargh S (2007). Getting to the site of inflammation: the leukocyte adhesion cascade updated. Nat Rev Immunol.

[27] Chavakis E, Choi EY, Chavakis T (2009). Novel aspects in the regulation of the leukocyte adhesion cascade. Thromb Haemost.

[28] Wilhelmsen K, Farrar K, Hellman J (2013). Quantitative in vitro assay to measure neutrophil adhesion to activated primary human microvascular endothelial cells under static conditions. J Vis Exp.

[29] Terheggen-Lagro SW, Rijkers GT, van der Ent CK (2005). The role of airway epithelium and blood neutrophils in the inflammatory response in cystic fibrosis. J Cyst Fibros.

[30] Hynes RO (2002). Integrins: bidirectional, allosteric signaling machines. Cell.

[31] Chen Y, Junger WG (2012). Measurement of oxidative burst in neutrophils. Methods Mol Biol.

[32] Hack CE, De Groot ER, Felt-Bersma RJ, Nuijens JH, Strack Van Schijndel RJ, Eerenberg-Belmer AJ (1989). Increased plasma levels of interleukin-6 in sepsis. Blood.

[33] Remick DG, Bolgos G, Copeland S, Siddiqui J (2005). Role of Interleukin-6 in Mortality from and Physiologic Response to Sepsis. Infect Immun.

[34] Hudson LD, Milberg JA, Anardi D, Maunder RJ (1995). Clinical risks for development of the acute respiratory distress syndrome. Am J Respir Crit Care Med.

[35] Yan J, Li S (2014). The role of the liver in sepsis. Int Rev Immunol.

[36] Nussler AK, Wittel UA, Nussler NC, Beger HG (1999). Leukocytes, the Janus cells in inflammatory disease. Langenbecks Arch Surg.

[37] Deitch EA (2012). Gut-origin sepsis: evolution of a concept. Surgeon.

[38] Miksa M, Wu R, Dong W, Komura H, Amin D, Ji Y (2009). Immature dendritic cell-derived exosomes rescue septic animals via milk fat globule epidermal growth factor-factor VIII [corrected]. J Immunol.

[39] Qiang X, Li J, Wu R, Ji Y, Chaung W, Dong W (2011). Expression and characterization of recombinant human milk fat globule-EGF factor VIII. Int J Mol Med.

[40] Shah KG, Wu R, Jacob A, Molmenti EP, Nicastro J, Coppa GF (2012). Recombinant human milk fat globule-EGF factor 8 produces dose-dependent benefits in sepsis. Intensive Care Med.

[41] Wu R, Chaung WW, Zhou M, Ji Y, Dong W, Wang Z (2010). Milk fat globule EGF factor 8 attenuates sepsis-induced apoptosis and organ injury in alcohol-intoxicated rats. Alcohol Clin Exp Res.

[42] Miksa M, Amin D, Wu R, Ravikumar TS, Wang P (2007). Fractalkine-induced MFG-E8 leads to enhanced apoptotic cell clearance by macrophages. Mol Med.

[43] Miksa M, Amin D, Wu R, Jacob A, Zhou M, Dong W (2008). Maturation-induced down-regulation of MFG-E8 impairs apoptotic cell clearance and enhances endotoxin response. Int J Mol Med.

[44] Pierschbacher MD, Ruoslahti E (1987). Influence of stereochemistry of the sequence Arg-Gly-Asp-Xaa on binding specificity in cell adhesion. J Biol Chem.

[45] Moon C, Han JR, Park HJ, Hah JS, Kang JL (2009). Synthetic RGDS peptide attenuates lipopolysaccharide-induced pulmonary inflammation by inhibiting integrin signaled MAP kinase pathways. Respir Res.

[46] Wang B, Wan JY, Zhang L, Min S (2012). Synthetic RGDS peptide attenuates mechanical ventilation-induced lung injury in rats. Exp Lung Res.

[47] Matsuo S, Yang WL, Aziz M, Jacob A, Wang P (2013). Cyclic arginine-glycine-aspartate attenuates acute lung injury in mice after intestinal ischemia/reperfusion. Crit Care.

[48] Fondevila C, Shen XD, Moore C, Busuttil RW, Coito AJ (2005). Cyclic RGD peptides with high affinity for alpha5beta1 integrin protect genetically fat Zucker rat livers from cold ischemia/reperfusion injury. Transplant Proc.

[49] Fondevila C, Shen XD, Duarte S, Busuttil RW, Coito AJ (2009). Cytoprotective effects of a cyclic RGD peptide in steatotic liver cold ischemia and reperfusion injury. Am J Transplant.

[50] Ding X, Wang X, Zhao X, Jin S, Tong Y, Ren H (2015). RGD peptides protects against acute lung injury in septic mice through Wisp1-integrin beta6 pathway inhibition. Shock.

[51] El Kebir D, Filep JG (2013). Modulation of neutrophil apoptosis and the resolution of inflammation through β2 integrins. Front Immunol.

[52] Jorgensen VL, Nielsen SL, Espersen K, Perner A (2006). Increased colorectal permeability in patients with severe sepsis and septic shock. Intensive Care Med.

[53] Wu R, Dong W, Wang Z, Jacob A, Cui T, Wang P (2012). Enhancing apoptotic cell clearance mitigates bacterial translocation and promotes tissue repair after gut ischemia-reperfusion injury. Int J Mol Med.

